# The Development of Highly pH-Sensitive Bacterial Cellulose Nanofibers/Gelatin-Based Intelligent Films Loaded with Anthocyanin/Curcumin for the Fresh-Keeping and Freshness Detection of Fresh Pork

**DOI:** 10.3390/foods12203719

**Published:** 2023-10-10

**Authors:** Siyu Zhou, Nan Li, Haonan Peng, Xingbin Yang, Dehui Lin

**Affiliations:** 1 Shaanxi Engineering Laboratory for Food Green Processing and Safety Control, Shaanxi Key Laboratory for Hazard Factors Assessment in Processing and Storage of Agricultural Products, College of Food Engineering and Nutritional Science, Shaanxi Normal University, Xi’an 710062, China; 2 Key Laboratory of Applied Surface and Colloid Chemistry (Ministry of Education), School of Chemistry and Chemical Engineering, Shaanxi Normal University, Xi’an 710119, China

**Keywords:** gelatin, curcumin, anthocyanin, bacterial cellulose nanofibers, freshness detection

## Abstract

The aim of this study was to develop highly pH-sensitive bacterial cellulose nanofibers/gelatin-based intelligent films, where the intelligent films were loaded with different ratios (10:0, 0:10 2:8, 5:5 and 8:2, *w*/*w*) of curcumin:anthocyanin (Cur/ATH), and the characterization of intelligent films was investigated. The results showed that the microstructures of intelligent films were much rougher as the proportion of curcumin increased. FTIR results showed that anthocyanin and curcumin were fixed in gelatin matrix by hydrogen bonds. Moreover, XRD results showed that curcumin had a significant effect on the crystal structure of the films. Interestingly, films loaded with a Cur/ATH ratio of 5:5 had the best mechanical and antioxidant properties and a high pH-sensitivity property. Consequently, the bacterial cellulose nanofibers/gelatin-based intelligent films loaded with a Cur/ATH ratio of 5:5 were used for the packaging of fresh pork, displaying good fresh-keeping and freshness detection effects. Therefore, this study suggested that bacterial cellulose nanofibers/gelatin-based intelligent films have great potential in the fresh-keeping and freshness detection of meat.

## 1. Introduction

Natural biopolymers such as proteins and polysaccharides have been widely used in the field of food packaging because of their good biocompatibility and good degradation performance [1]. Gelatin is an animal protein obtained by partial hydrolysis of collagen. It has many advantages, including low cost, degradability, high transparency, good biocompatibility and good film-forming performance [2]. However, the high hydrophilicity of gelatin leads to its poor water resistance and mechanical properties [3]. It has been demonstrated that the performance of gelatin can be improved by adding nanofillers. Bacterial cellulose nanofibers (BCNs) are a new type of natural material that can be used as a reinforcing agent for biological composites. They have good biocompatibility and can improve the water-holding capacity, mechanical properties, thermal stability and other functional properties of a polymer [4]. However, the nanofiber composite films prepared based on biopolymers have poor antioxidant and antibacterial properties and no indicative function.

With the increasing improvement in living standards, the demand for safe and fresh food is growing. Therefore, it is very important to develop intelligent packaging with the functions of monitoring the food conditions and informing consumers about the quality of packaged food in real time [5,6]. It has been reported that some natural pigments have pH sensitivity in addition to their antioxidant and antibacterial properties. For this reason, some natural pigments can be used as pH indicators in food packaging because the pH values of the microenvironment inside the package will change as a result of some chemical substances from the packaged food during food storage. Therefore, food package films loaded with pH-sensitive natural pigments not only have a fresh-keeping property, but also have a freshness detection property [7,8]. Anthocyanin (ATH)is a natural water-soluble flavonoid pigment based on polyphenols [9] and has excellent antibacterial, antioxidant and pH sensitivity properties [10]. Curcumin (Cur) is a natural polyphenol fat-soluble substance obtained from the rhizome of turmeric and has pH sensitivity, anti-inflammatory, antibacterial and antioxidant properties. Moreover, it has been demonstrated that anthocyanin and curcumin are non-toxic, and they have been widely used in the field of food [11,12,13,14]. Therefore, anthocyanin and curcumin can also be used as freshness indicators for meat. It has been reported that the pH sensitivity of mixed natural pigments is higher than that of a single natural pigment [15,16,17], because some natural pigments (e.g., anthocyanin) are only sensitive to acidic conditions, whilesome natural pigments (e.g., curcumin) are only sensitive to alkaline conditions [18]. Therefore, it is necessary to improve the sensitivity of intelligent films loaded with mixed natural pigments.

Hence, bacterial cellulose nanofibers/gelatin-based intelligent films loaded with different ratios of curcumin and anthocyanin were developed to improve the pH sensitivity of the films in the present study. The color changes of mixed natural pigments at different pH values and the microstructure, physical properties, barrier properties, mechanical properties, color, transmittance and opacity of bacterial cellulose nanofibers/gelatin-based intelligent films loaded with different pigment proportions were investigated. Moreover, the antioxidant and pH sensitivity properties of the prepared intelligent films were also studied. Finally, the indication effect of intelligent films on pork was investigated.

## 2. Materials and Methods

### 2.1. Materials

Food-grade gelatin (Gel) was supplied by Beijing Solarbio Science & Technology Co., Ltd. (Beijing, China). Food-grade glycerol, curcumin (purity 95%), anthocyanin from blueberry extract (purity 35%), NaOH, HCl and 2,2-diphenyl-1-picryl hydrazine (DPPH) were purchased from Xi’an Senbo Biology Co., Ltd. (Xi’an, China). *Komagataeibacter hansenii* CGMCC 3917 was donated by the Fermentation Technology Innovation Laboratory of Northwest A&F University (Yanglin, China).

### 2.2. Production of Bacterial Cellulose Nanofibers (BCNs)

BCNs were prepared according to our previous method [19]. *Komagataeibacter hansenii* CGMCC 3917 was inoculated at pH 5.0 with K_2_HPO_4_ (0.1%, *w*/*v*), yeast extract (0.5%, *w*/*v*), MgSO_4_ (1.5%, *w*/*v*), glucose (2%, *w*/*v*) and ethanol (2%, *w*/*v*) and statically cultured at 30 °C for 14 days. The harvested bacterial cellulose (BC) was boiled for 30 min in a NaOH solution and then washed with running water to neutral. Pure BC was obtained, and the wet bacterial cellulose was crushed with a stirrer to obtain an aqueous bacterial cellulose solution, followed by centrifugation. Then, the BC suspension was hydrolyzed for 8 h at 80 °C with 3M HCl and centrifuged to neutral. Finally, the BCNs were obtained and stored in a refrigerator at 4 °C [20].

### 2.3. Preparation of Bacterial Cellulose Nanofibers/Gelatin-Based Intelligent Films

First, 6% (*w*/*v*) gelatin and 20% (*w*/*w*, based on gelatin) glycerol were dissolved in distilled water at 95 °C and stirred until completely dissolved. Then, 2% BCNs (*w*/*w*, based on gelatin) were mixed with a high-shear blender at 8000 rpm/min for 2 min to obtain a gelatin film-forming solution. Then, 3% (*w*/*w*, based on gelatin) pigments (Cur/ATH, 10:0, 0:10 2:8, 5:5 and 8:2, *w*/*w*) were added to the film-forming solutions. Then, the film-forming solutions were mixed with a high-shear blender at 8000 rpm/min for 2 min, poured into Petri dishes, and dried in a drying oven at 30 °C for 24 h. The film was vacuum-packed and stored at room temperature in the dark. The films were recorded as Cur/ATH_10:0_, Cur/ATH_0:10_, Cur/ATH_2:8_, Cur/ATH_5:5_ and Cur/ATH_8:2_.

### 2.4. UV–Vis Absorption Spectra of Natural Pigment Solutions at Different pH Values

First, 0.1 mL of natural pigment solution (1 g/L) was added to 10 mL of buffer solution with different pH values (pH = 1, 3, 5, 7, 9, 11 and 13). The spectra of natural pigment solutions at different pH values were scanned by a UV-Vis spectrophotometer (UV300, InsMark, Shanghai, China), and the scanning range was 300–800 nm [18].

### 2.5. Scanning Electron Microscopy (SEM)

The samples were adhered to an aluminum plate and were plated with gold by a sputter coater for 60 s under a 20 kV accelerating voltage. The microstructure of the films was determined by a scanning electron microscope (S-3400N, HITACHI, Tokyo, Japan).

### 2.6. Atomic Force Microscopy (AFM)

The surface morphology of the films was characterized by atomic force microscopy (AFM, Bruker, Billerica, MA, USA). Firstly, the film was cut into a 10 × 10 mm^2^ square and fixed on the silicon wafer with double-sided adhesive. The AFM image of the air side of the film was obtained in the tapping mode. The surface roughness parameters (root-mean-square roughness Rq and average roughness Ra) of the films were obtained by NanoScope Analysis software 2.0.

### 2.7. Fourier Transform Infrared (FTIR) Spectroscopy

The spectra of samples were recorded by a FTIR spectrometer (BRUKER, INVENNIO S, Karlsruhe, Germany) at 4000–400 cm^−1^ with a resolution of 4 cm^−1^ [21].

### 2.8. X-ray Diffraction (XRD)

XRD images of films were obtained by X-ray diffraction. All samples were scanned at room temperature by an X-ray diffractometer with Kα Cu X-ray radiation at a scanning angle of 5° to 40° with an interval angle of 0.02° [22].

### 2.9. Water Contact Angle (WCA)

The water contact angle of the films was tested by an optical contact angle measuring instrument (theta flex, Biolin Scientific, Göteborg, Sweden). For this test, 2 μL of MilliQ water was lightly dropped onto the films, and photos were recorded with a high-speed camera.

### 2.10. Water Content (WC) and Water Solubility (WS)

The initial weight *m* (g) of the film samples (2 × 2 cm^2^) was recorded. Then, the film samples were dried in an oven for 24 h at 105 °C, and the weight *M* (g) of the film samples was recorded [23]. The *WC* of films was calculated as follows:

$$WC=\frac{m-M}{m}\times 100\%$$

The weight of the film samples after drying for 24 h at 105 °C was denoted as *M* (g). The dry samples were immersed in a beaker containing 15 mL of distilled water and stored for 24 h at 25 °C. Then, the impregnation solutions were poured out, and the films were placed in the oven at 105 °C for 24 h; the weight of the samples was recorded as *W* (g) [24], and then the *WS* of the films was calculated as follows:

$$WS=\frac{M-W}{M}\times 100\%$$

### 2.11. Thickness, Water Vapor Permeability (WVP) and Relative Oxygen Transmission Rate (ROT)

The thickness of the films was measured by a digital caliper (Minsks Co. Ltd., Xi’an, China), and 10 random positions were selected. Water vapor permeability (WVP) was measured according to our previous method [4], and a simple modification was made [18]. A 50 mL centrifugal tube containing 30 mL of distilled water was coated with films. The centrifugal tube was placed in a desiccator containing silica for 12 h and then weighed. The calculation formula for water vapor permeability was as follows:

$$WVP[g\cdot m\cdot (m^{2}\cdot s\cdot {\mathrm{Pa})}^{-1}]=\frac{\Delta m\times d}{A\times t\times \Delta p}$$

where ∆*m* is the weight difference of the centrifugal tubes, *d* is the thickness of the films, *A* is the effective area of the films, ∆*p* is the water vapor pressure difference on both sides of the films (3.168 × 10^3^ Pa, 25 °C) and *t* is time.

The relative oxygen transmission rate (ROT) was determined according to the method of Zhang et al. [25]. In short, 3 g of deoxidizer (reduced iron powder: activated carbon: sodium chloride = 0.5:1:1.5) was added to the centrifugal tube, and the film was placed above the centrifugal tube and put into a dryer at 25 °C. A saturated solution of barium chloride (RH, 90%) was placed at the bottom of the desiccator. After drying for 48 h, the final weight of the centrifugal tube was recorded, and the relative oxygen transmission rate of the films was calculated as follows:

$$\mathrm{ROT}[g\cdot (m^{2}\cdot s{)}^{-1}]=\frac{m_{f}-m_{i}}{t\times A}$$

where *m_f_* is the final weight of the centrifugal tubes after 48 h, *m_i_* is the initial weight of the centrifugal tubes, *A* is the effective area of the films and *t* is time.

### 2.12. Mechanical Properties

The mechanical properties were tested according to our previous method with some modifications [4]. The tensile strength (TS) and elongation at break (EAB) of the samples were determined by a universal material testing machine (China xi’an Minsk Co., Ltd., Xi’an, China). The film samples were cut into 2 × 8 cm^2^ pieces; the initial grip separation was set as 50 mm, and the cross-head speed was set as 30 mm/min. The tensile strength (TS) and elongation at break (EAB) were calculated as follows:

$$\mathrm{TS}(\mathrm{MPa})=\frac{N}{a\times b}$$

$$\mathrm{EAB}(\%)=\frac{L-L_{0}}{L_{0}}\times 100$$

where *N* is the maximum fracture force of the samples (N), *a* is the width of the samples (mm), *b* is the thickness of the samples (mm), *L*_0_ is the initial length of the samples (mm) and *L* is the final length of the samples (mm).

### 2.13. Transmittance and Opacity Measurements

The films were cut into 4 × 1 cm^2^ pieces and were measured by a UV300 spectrophotometer in the range of 300–800 nm, and air was used as a reference. In addition, the opacity of the films was determined at 600 nm [19], and was calculated as follows:

$$\mathrm{Opacity}=\frac{A_{600}}{X}$$

where *A*_600_ is the absorbance of film at 600 nm and *X* is the film thickness (mm).

### 2.14. Colorimetric Analysis

The colorimetric analysis of films was measured by a colorimeter (3nh Co. Ltd., NR100, Shenzhen, Chain), and the L* value, a* value and b* value were used to evaluate the color of the intelligent films. The total color difference ∆E* was calculated as follows:

$$∆E^{*}=\sqrt{{\left(L^{*}-L_{0}^{*}\right)}^{2}+{\left(a^{*}-a_{0}^{*}\right)}^{2}+{\left(b^{*}-b_{0}^{*}\right)}^{2}}$$

where *L*_0_*, *a*_0_* and *b*_0_* are the color parameter values of a standard white board and L*, a* and b* are the color parameter values of the bacterial cellulose nanofibers/gelatin-based intelligent films.

### 2.15. Determination of Antioxidant Property

The DPPH radical-scavenging activity was measured according to the method of Ma et al. [26]. The specific operation was as follows: First, 0.1 g of the film was dissolved in 5 mL of ethanol aqueous solution and stirred for 30 min. Then, 1 mL of the sample solution was mixed with 3 mL of a DPPH ethanol solution (50 mg/L) and then placed for 30 min in the dark. The absorbance of the solution at 517 nm was measured by a UV–visible spectrophotometer. The DPPH radical-scavenging activity of the film samples was calculated as follows:

$$\mathrm{DPPH}\;\mathrm{scavenging}\;\mathrm{activity}(\%)=100\times \frac{A_{\mathrm{sample}}}{A_{\mathrm{DPPH}}}$$

where *A_DPPH_* is the absorbance of the control and *A_sample_* is the absorbance of the sample solution.

### 2.16. pH Sensitivity of Intelligent Films

The films were cut into 2 × 2 cm^2^ pieces and immersed in solutions with different pH values (pH = 1, 3, 5, 7, 9, 11 and 13), and the sensitivity to different pH values was tested by observing the color changes of the bacterial cellulose nanofibers/gelatin-based intelligent films.

### 2.17. The Response of the Intelligent Films to Volatile Ammonia

The response of the intelligent films to volatile ammonia was determined according to a reported method with minor modifications [27]. First, 40 mL of ammonia (0.1 mol/L) was put into a 50 mL sealed beaker, and the films were placed above the ammonia solution (1 cm). The samples were placed under a standard light source at 25 °C, and the a*, b* and ∆E* values of the films were recorded every 2 min for a total of 20 min.

### 2.18. Application of the Bacterial Cellulose Nanofibers/Gelatin-Based Intelligent Films in the Packaging of Pork

The bacterial cellulose nanofibers/gelatin-based intelligent films loaded with a Cur/ATH ratio of 5:5 were used for the packaging of pork to investigate the fresh-keeping and freshness detection properties of the films. Fresh pork was packaged with bacterial cellulose nanofibers/gelatin-based films and bacterial cellulose nanofibers/gelatin-based intelligent films, and fresh pork without packaging was used as a blank. All samples were placed in a refrigerator at 4 °C for further determination.

The total volatile basic nitrogen (TVB-N) of pork was determined by Kjeldahl apparatus (Dimension Icon, Bruker, Billerica, MA, USA), and was expressed as mg/100 g. To determine the pH of pork, 5 g of pork and 20 mL of distilled water were mixed with a high-shear blender at 10,000 rpm for 10 min, and the pH values of the samples were measured by a pH meter. The hardness and springiness of pork were determined by a structural analyzer. The parameters were set as follows: probe P36R, trigger force 5 g, probe speed 1.0 mm/s, test interval 5 s. The color change of films could directly reflect the quality of the packaged pork. A colorimeter was used for the colorimetric analysis of all the samples. Meanwhile, all the samples were recorded with a camera.

### 2.19. Statistical Analysis

Analysis of variance (ANOVA) was performed by SPSS 22.0 software. All the data were expressed as standard ± deviation. The comparison of the mean values between two groups was performed using the least significant difference (LSD) with a confidence interval of 95 percent. The results were drawn by Origin 2021 software.

## 3. Results and Discussion

### 3.1. UV–Vis Spectra of Pigment Solutions at Different pH Values

**Figure 1:**
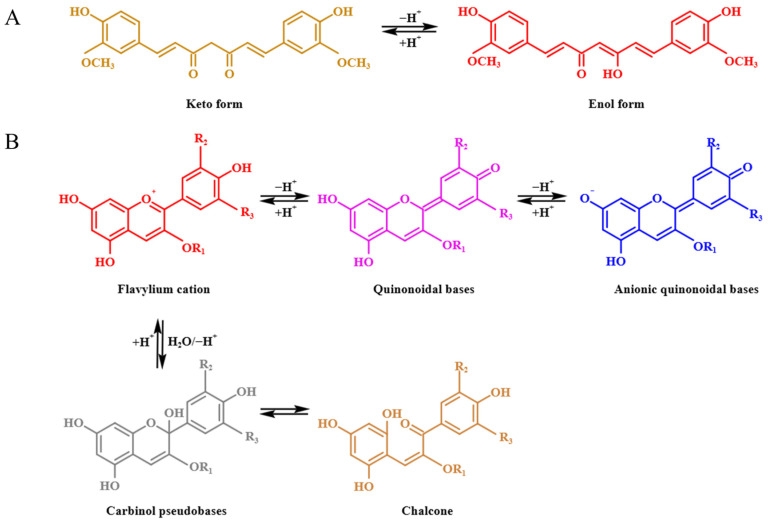
Structural variation of curcumin (**A**) and anthocyanin (**B**) at different pH values.

**Figure 2:**
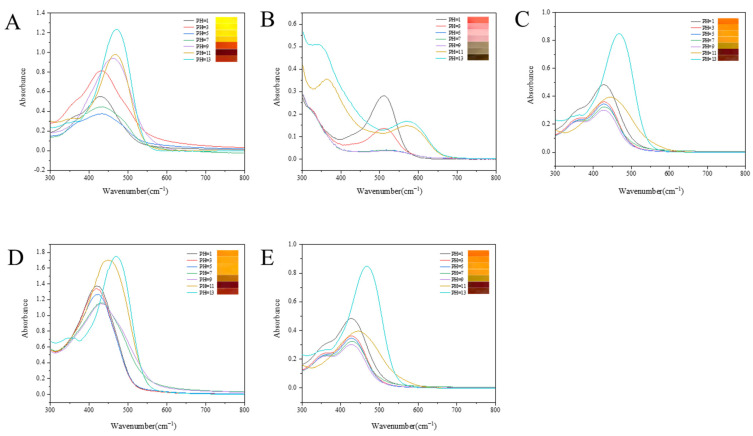
Color and visible spectra of Cur (**A**), ATH (**B**), Cur/ATH = 2:8 (**C**), Cur/ATH = 5:5 (**D**) and Cur/ATH = 8:2 (**E**) at pH =1, 3, 5, 7, 9, 11 and 13.

The schematics of the change in structure of curcumin and anthocyanin in different pH values are shown in Figure 1. As shown in Figure 2A, the color of the curcumin solution was almost yellow at pH ≤ 7, showing that the curcumin solution was not highly sensitive to the pH values as pH ≤ 7, while at pH > 7, the color of the curcumin solution turned orange-red (pH = 9), chocolate (pH = 11) and vermilion (pH = 13), respectively, displaying different kinds of colors at different pH values, which suggested that the curcumin solution was sensitive to pH values at pH > 7. Meanwhile, the corresponding spectra of the curcumin solution were determined at different pH values, and the maximum absorption wavelength of the curcumin solution shifted from 428 nm to 432 nm, 434 nm, 436 nm, 462 nm, 467 nm and 472 nm as the pH value increased from 1 to 3, 5, 7, 9, 11 and 13, respectively. This result was probably due to the keto enol tautomerism transformation of curcumin [28]. Under acidic and neutral conditions, curcumin was mainly composed of the keto type, and its color was yellow. Under alkaline conditions, curcumin was mainly composed of the enol type, and its color changed from yellow to red and gradually deepened with the increase in pH values (Figure 1A) [29,30]. Figure 2B shows that the color of anthocyanin solution at different pH values; anthocyanin was tangerine, pale pink, pink, pink gray, light brown, brown and dark brown when the pH value was 1, 3, 5, 7, 9, 11 and 13, respectively. Meanwhile, the corresponding maximum absorption wavelength of the anthocyanin solution moved from 512 nm to 516 nm, 519 nm, 521 nm, 543 nm, 569 nm and 575 nm as the pH value increased from 1 to 3, 5, 7, 9, 11 and 13, respectively, which suggesting that with the increase of the pH values, the corresponding structure of anthocyanin was changed, as displayed in Figure 1B [31].

In order to improve the pH sensitivity of the pigment solutions, different ratios of Cur/ATH solutions were investigated in the present study. As displayed in Figure 2C, the color of the mixture solution with a Cur/ATH ratio of 2:8 changed from jacinth to brown and the corresponding maximum absorption wavelength shifted from 428 nm to 472 nm as the pH value changed from 1 to 13. As shown in Figure 2D, the color of the mixture solution with a Cur/ATH ratio of 5:5 changed from orange to brown and the corresponding maximum absorption wavelength moved from 427 nm to 467 nm as the pH value changed from 1 to 13. As displayed in Figure 2E, the color of the mixture solution with a Cur/ATH ratio of 8:2 changed from turmeric to brown and the corresponding maximum absorption wavelength shifted from 418 nm to 468.5 nm as the pH value changed from 1 to 13. The results showed that curcumin and anthocyanin had a pH sensitivity property and could be used as pH indicators in intelligent packaging.

### 3.2. Microstructure of Bacterial Cellulose Nanofibers/Gelatin-Based Intelligent Films

**Figure 3:**
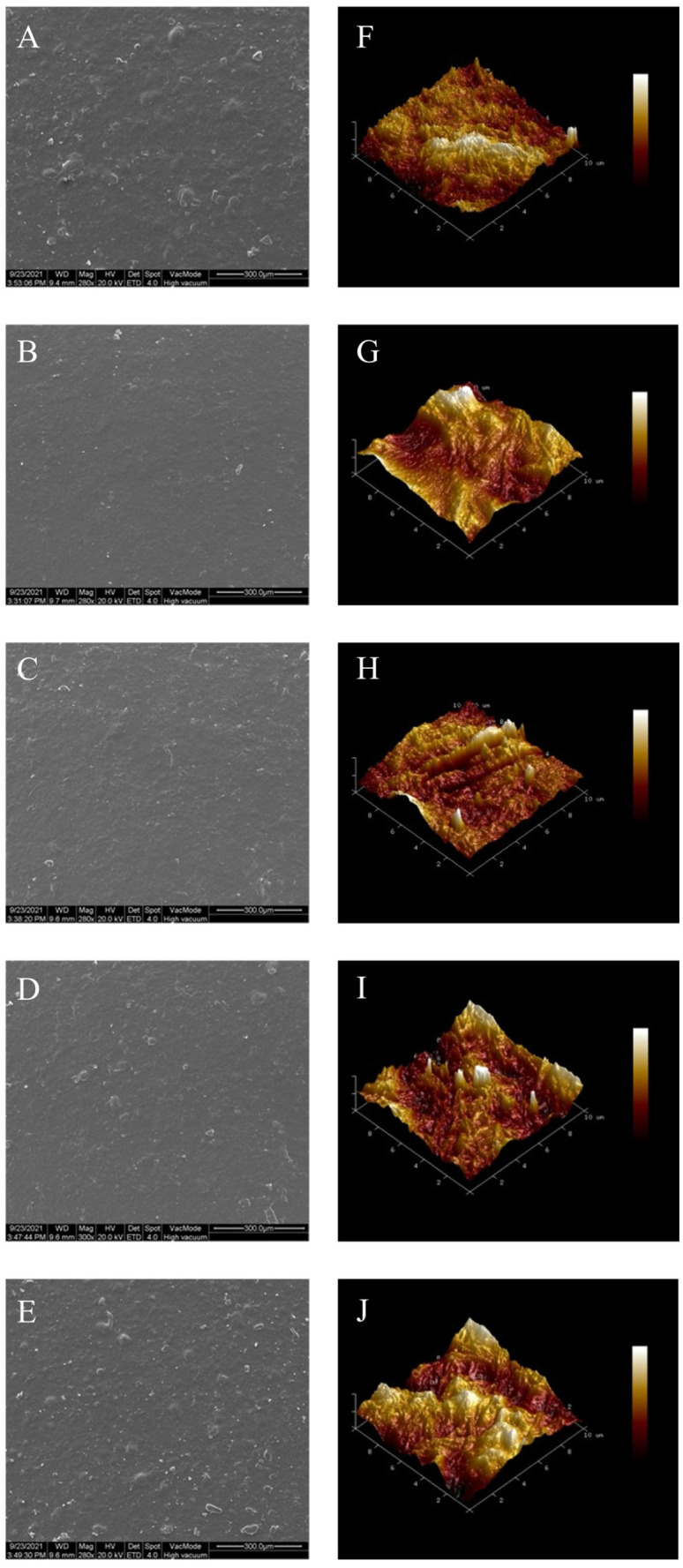
SEM images of bacterial cellulose nanofibers/gelatin-based intelligent films with different natural pigments: Cur/ATH_10:0_ (**A**), Cur/ATH_0:10_ (**B**), Cur/ATH_2:8_ (**C**), Cur/ATH_5:5_ (**D**), Cur/ATH_8:2_ (**E**); AFM images: Cur/ATH_10:0_ (**F**), Cur/ATH_0:10_ (**G**), Cur/ATH_2:8_ (**H**), Cur/ATH_5:5_ (**I**), Cur/ATH_8:2_ (**J**).

SEM images of the bacterial cellulose nanofibers/gelatin-based intelligent films are shown in Figure 3A–E. The surface of the bacterial cellulose nanofibers/gelatin-based intelligent film loaded with only curcumin was the roughest and most uneven as compared with the other four types of intelligent films, which was probably due to curcumin being fat-soluble, leading to the aggregation in the bacterial cellulose nanofibers/gelatin-based intelligent films. The microstructure of the bacterial cellulose nanofibers/gelatin-based intelligent film loaded with only anthocyanin was the smoothest and flattest, which was likely attributed to the good dispersion of anthocyanin in bacterial cellulose nanofibers/gelatin-based intelligent films as a result of its water solubility. For the bacterial cellulose nanofibers/gelatin-based intelligent films loaded with curcumin and anthocyanin, as the ratio of Cur/ATH increased, the surface of the films became rougher and more uneven, which was probably because anthocyanin could dissolve in water and disperse more fully in the gelatin matrix, while curcumin would precipitate and accumulate randomly in the gelatin matrix in the process of drying [32].

Figure 3F–J show the surface topography of the bacterial cellulose nanofibers/gelatin-based intelligent films loaded with different ratios of Cur/ATH as observed by atomic force microscopy; the surface of the bacterial cellulose nanofibers/gelatin-based intelligent films became rougher as the ratio of Cur/ATH increased, in agreement with the SEM results described in Figure 3A–E. Table 1 shows the root-mean-square roughness (Rq) and average roughness (Ra) of the intelligent films; the Rq and Ra values of the bacterial cellulose nanofibers/gelatin-based intelligent films loaded with only anthocyanin were the smallest, with the values of 16.70 nm and 13.15 nm, respectively. With the increase in Cur/ATH ratios, the Rq and Ra values of the bacterial cellulose nanofibers/gelatin-based intelligent films increased. The Rq and Ra of bacterial cellulose nanofibers/gelatin-based intelligent films loaded with only curcumin displayed the highest values, 52.75 nm and 41.9 nm, respectively, which was in line with the SEM results described above.

### 3.3. Fourier Transfors Infrared (FTIR) Spectroscopy

**Figure 4:**
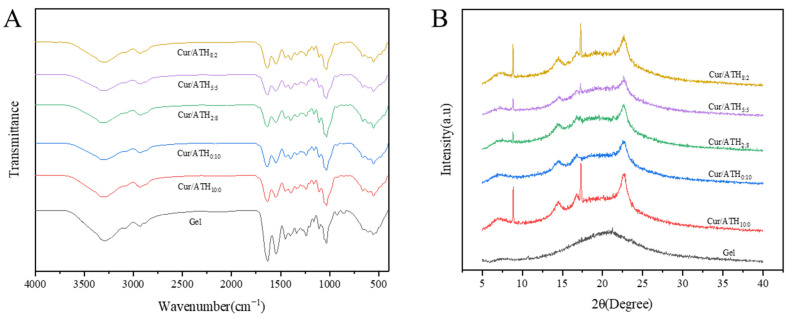
FTIR spectra (**A**) and X-ray patterns (**B**) of the bacterial cellulose nanofibers/gelatin-based intelligent films with different ratios of natural pigments.

The FTIR spectra of the bacterial cellulose nanofibers/gelatin-based intelligent films are displayed in Figure 4A. The wide absorption band in the pure bacterial cellulose nanofibers/gelatin-based film in the range of 3709–3005 cm^−1^ was related to the free stretching vibration of the hydroxyl group, and the peak observed at 3293 cm^−1^ was due to the presence of the hydroxyl group, which is consistent with the results reported in the literature [31]. The characteristic peak of the wavelength at 2932 cm^−1^ was C-H tensile vibration, the peak at 1634 cm^−1^ was attributed to the carbonyl stretching, the peak at 1549 cm^−1^ was due to N-H stretching and the peak at 1033 cm^−1^ was due to C-O stretching [33,34]. The results showed that all the bacterial cellulose nanofibers/gelatin-based films had similar main peaks, while the band at 3293 cm^−1^ (pure gelatin films) moved to 3298 cm^−1^ (films loaded with a Cur/ATH ratio of 10:0) and 3300 cm^−1^ (films loaded with a Cur/ATH ratio of 0:10), indicating that the new hydrogen bonds were formed and thus confirming that anthocyanin and curcumin were successfully combined into the gelatin matrix [28].

### 3.4. X-ray Diffraction (XRD) Analysis

Figure 4B shows the XRD pattern of bacterial cellulose nanofibers/gelatin-based intelligent films. All films had diffraction peaks near 14.48°, 16.77° and 22.53°, which corresponded to the (1−10), (110) and (200) crystallographic planes of native cellulose I, respectively. Additionally, for the intelligent films loaded with curcumin, diffraction peaks also appeared at 8.86° and 17.30°, and the intensity of the diffraction peaks increased significantly as the proportion of curcumin in the total pigments increased. The results showed that curcumin had a significant effect on the crystal structure of the bacterial cellulose nanofibers/gelatin-based intelligent films, in agreement with our previous reports [4].

### 3.5. Water Contact Angle (WCA)

**Figure 5:**
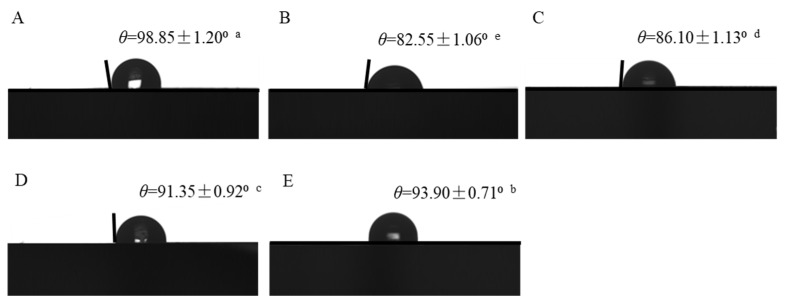
The contact angle of bacterial cellulose nanofibers/gelatin-based intelligent films with different ratios of natural pigments: Cur/ATH_10:0_ (**A**), Cur/ATH_0:10_ (**B**), Cur/ATH_2:8_ (**C**), Cur/ATH_5:5_ (**D**), Cur/ATH_8:2_ (**E**). Different letters (a, b, c, d and e) differ significantly on the same line.

The contact angle can reflect the surface hydrophilicity and hydrophobicity of the films. As shown in Figure 5, the water contact angle of the bacterial cellulose nanofibers/gelatin-based intelligent films ranged from 80° to 100°. The contact angle of the bacterial cellulose nanofibers/gelatin-based intelligent films loaded with only curcumin had the highest value (98.85°), while the contact angle of the bacterial cellulose nanofibers/gelatin-based intelligent films loaded with only anthocyanin had the lowest value (82.55°). Moreover, the contact angles of the films loaded with the Cur/ATH ratios of 2:8, 5:5 and 8:2 were 86.10°, 91.35° and 93.90°, respectively, indicating that curcumin could increase the surface hydrophobicity of the bacterial cellulose nanofibers/gelatin-based intelligent films to make intelligent films better meet the needs of food packaging [35].

### 3.6. Thickness, Water Content (WC) and Water Solubility (WS) of Intelligent Films

The thickness of the bacterial cellulose nanofibers/gelatin-based intelligent films is shown in Table 2. The bacterial cellulose nanofibers/gelatin-based intelligent films loaded with only anthocyanin had the lowest thickness (0.026 mm), while the bacterial cellulose nanofibers/gelatin-based intelligent films loaded with only curcumin had the highest thickness (0.030 mm). As the proportion of curcumin increased, the thickness of the films increased to 0.027 mm (films loaded with a Cur/ATH ratio of 2:8), 0.028 mm (films loaded with a Cur/ATH ratio of 5:5) and 0.028 mm (films loaded with a Cur/ATH ratio of 8:2). The water content (WC) and water solubility (WS) of bacterial cellulose nanofibers/gelatin-based intelligent films are shown in Table 2. The bacterial cellulose nanofibers/gelatin-based films loaded only with anthocyanin had the highest WC value (20.82%) as compared with those films loaded with curcumin. This result was due to the fact that anthocyanin contains a large number of hydrophilic groups, exhibiting good water retention. As the proportion of curcumin in total pigment increased, the WC values of the bacterial cellulose nanofibers/gelatin-based intelligent films decreased to 17.32% (films loaded with a Cur/ATH ratio of 2:8), 16.88% (films loaded with a Cur/ATH ratio of 5:5), 14.31% (films loaded with a Cur/ATH ratio of 8:2) and 8.79% (films loaded with a Cur/ATH ratio of 10:0), which was because the addition of curcumin increased the hydrophobic properties of the bacterial cellulose nanofibers/gelatin-based intelligent films, in agreement with a previous result reported in the literature [36]. For the water solubility of the bacterial cellulose nanofibers/gelatin-based intelligent films, a similar trend was found. The bacterial cellulose nanofibers/gelatin-based films loaded with only curcumin had the lowest value of WS (49.12%). The WS values of the films loaded with curcumin decreased gradually as the proportion of curcumin increased, suggesting that curcumin could improve the water resistance of the films, in agreement with previous reports [37].

### 3.7. Water Vapor Permeability (WVP) and Relative Oxygen Transmission Rate (ROT) of Intelligent Films

Water vapor permeability (WVP) and relative oxygen transmission rate (ROT) are important indices of food packaging; thus, the WVP and ROT were investigated in the present study. As shown in Table 2, there were significant differences in the WVP values of bacterial cellulose nanofibers/gelatin-based intelligent films (*p* < 0.05): bacterial cellulose nanofibers/gelatin-based intelligent films loaded with only anthocyanin had the highest WVP value (2.67 × 10^−10^ g∙m/m^2^∙s∙Pa) as compared with the other four types of intelligent films, and the WVP values of films loaded with curcumin decreased to 2.57 × 10^−10^ g∙m/m^2^∙s∙Pa (films loaded with a Cur/ATH ratio of 2:8), 2.37 × 10^−10^ g∙m/m^2^∙s∙Pa (films loaded with a Cur/ATH ratio of 5:5), 2.09 × 10^−10^ g∙m/m^2^∙s∙Pa (films loaded with a Cur/ATH ratio of 8:2) and 2.22 × 10^−10^ g∙m/m^2^∙s∙Pa (films loaded with a Cur/ATH ratio of 10:0), which wasprobably due the hydrophobic properties of curcumin and the dispersion of curcumin in the gelatin matrix for a certain proportion of curcumin, resulting in the migration path of water molecules in the films was lengthened [32]. As shown in Table 2, as the proportion of curcumin in total pigments increased, the ROT value decreased from 2.81 × 10^−3^ g/m^2^∙s (films loaded with only anthocyanin) to 2.44 × 10^−3^ g/m^2^∙s (films loaded with a Cur/ATH ratio of 5:5) and then increased to 2.96 × 10^−3^ g/m^2^∙s (films loaded with only curcumin). The results suggested that the low addition of curcumin could improve the oxygen-blocking property of the intelligent films, which was likely due to the presence of the interaction between curcumin and gelatin that would lengthen the oxygen infiltration. The dispersion of high addition of curcumin in the film was not uniform, thus decreasing the oxygen barrier property [18].

### 3.8. Mechanical Properties

Tensile strength (TS) and elongation at break (EAB) are important indicators for the evaluation of mechanical properties. As shown in Table 3, the TS values displayed significant differences (*p* < 0.05): the bacterial cellulose nanofiber/gelatin-based intelligent film loaded with only curcumin was the lowest (45.03 Mpa) as compared with the other four types of intelligent films, which was probably because the high curcumin content damaged the structure of the films and resulted in a rougher and looser internal structure of the films as a result of its poor dispersion, in agreement with the results reported in the literature [38]. As the proportion of anthocyanin in total pigments increased, the TS value increased from 45.03 MPa (films loaded with a Cur/ATH ratio of 10:0) to 49.06 Mpa (films loaded with a Cur/ATH ratio of 8:2) and 62.96 Mpa (films loaded with a Cur/ATH ratio of 5:5) and then decreased to 57.01 MPa (films loaded with a Cur/ATH ratio of 2:8) and 52.71 MPa (films loaded with a Cur/ATH ratio of 0:10), which suggested that withina certain proportion of anthocyanin, anthocyanin could be well distributed in the gelatin matrix due to the interactions of hydrogen bonds and result in significant interfacial contact, thus increasing the TS values [39].

The EAB value of the bacterial cellulose nanofibers/gelatin-based intelligent film loaded with only curcumin was the lowest (13.25%), which was due to the poor dispersion of curcumin prevented the relative movement of molecules. As the proportion of anthocyanin in total pigments increased, the EAB value increased from 13.25% (films loaded with a Cur/ATH ratio of 10:0) to 14.73% (films loaded with a Cur/ATH ratio of 8:2) and 17.42% (films loaded with a Cur/ATH ratio of 5:5) and then decreased to 16.70% (films loaded with a Cur/ATH ratio of 2:8) and 15.30% (films loaded with a Cur/ATH ratio of 0:10), indicating that the EAB values of the films were significantly affected by the proportion of anthocyanin and curcumin. In conclusion, the mechanical properties of films loaded with a Cur/ATH ratio of 5:5 were better than those of the other films. The results showed that the combination of curcumin and anthocyanin was an effective way to improve the mechanical properties of bacterial cellulose nanofibers/gelatin-based intelligent films, in agreement with previous reports [40].

### 3.9. Color and Transparency Properties of Bacterial Cellulose Nanofibers/Gelatin-Based Intelligent Films

Table 4 shows the color parameters of the films (L*, a*, b* and ΔE*). There was no significant difference in L* value for all films (*p* > 0.5). For the films loaded with two pigments, as the proportion of curcumin in total pigments increased, the a* value of the intelligent films increased from 0.39 (films loaded with a Cur/ATH ratio of 2:8) to 3.28 (films loaded with a Cur/ATH ratio of 8:2), the b* value increased from 48.37 (films loaded with a Cur/ATH ratio of 2:8) to 59.48 (films loaded with a Cur/ATH ratio of 8:2) and the ΔE* value increased from 46.72 (films loaded with a Cur/ATH ratio of 2:8) to 57.72 (films loaded with a Cur/ATH ratio of 8:2), showing that the color characteristics of films depended on the color of curcumin and anthocyanin, in agreement with the appearance of films.

**Figure 6:**
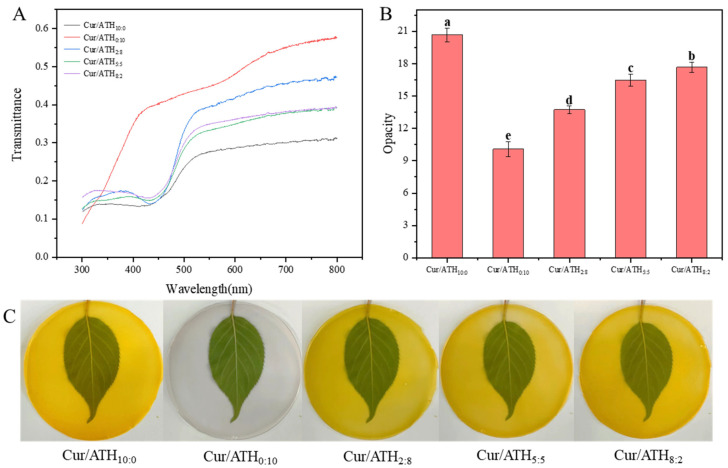
Light transmittance (**A**), opacity (**B**) and digital images (**C**) of bacterial cellulose nanofibers/gelatin-based intelligent films with different ratios of natural pigments. Different letters (a, b, c, d and e) differ significantly on the same line.

As shown in Figure 6A, bacterial cellulose nanofibers/gelatin-based intelligent films loaded with only curcumin had the lowest transmittance (31.12%), while bacterial cellulose nanofibers/gelatin-based intelligent films loaded with only anthocyanin had the highest transmittance (57.54%). For films loaded with two types of pigments, as the proportion of curcumin in total pigments increased, the light transmittance decreased gradually. In addition, the transmittance of all intelligent films at 350 nm was lower than 20%, indicating that intelligent films could effectively block ultraviolet light and prevent food from deterioration due to oxidation caused by light [28]. The change tendency of the opacity of the intelligent films is shown in Figure 6B. The bacterial cellulose nanofibers/gelatin-based intelligent films loaded with only anthocyanin had the lowest opacity, while the bacterial cellulose nanofibers/gelatin-based intelligent films loaded with only curcumin had the highest opacity, in agreement with the change tendency of transmittance described above and the appearance of the intelligent films displayed in Figure 6A,C.

### 3.10. Antioxidant Property of Bacterial Cellulose Nanofibers/Gelatin-Based Intelligent Films

**Figure 7:**
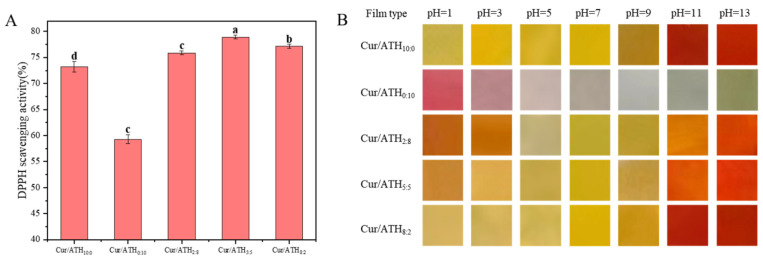
DPPH radical-scavenging activity (**A**) and color response at different pH values (**B**) of bacterial cellulose nanofibers/gelatin-based intelligent films with different ratios of natural pigments. Different letters (a, b, c and d) differ significantly on the same line.

Curcumin and anthocyanin have good antioxidant activity, which is mainly because the phenolic hydroxyl group can bind with free radicals and provide a hydrogen atom [14]. As shown in Figure 7A, the DPPH free-radical-scavenging rate of bacterial cellulose nanofibers/gelatin-based intelligent films loaded with only curcumin was 73.21%, indicating that curcumin had strong antioxidant activity, in agreement with previous results reported in the literature [31]. The DPPH radical-scavenging rate of bacterial cellulose nanofibers/gelatin-based intelligent films loaded with only anthocyanin was 59.28%, indicating that anthocyanin had antioxidant capacity. Compared with bacterial cellulose nanofibers/gelatin-based intelligent films loaded with only anthocyanin, the antioxidant capacity of the films loaded with curcumin and anthocyanin were significantly increased to 75.87% (films loaded with a Cur/ATH ratio of 2:8), 78.91% (films loaded with a Cur/ATH ratio of 5:5) and 77.17% (films loaded with a Cur/ATH ratio of 8:2). This result suggested that anthocyanin and curcumin had a synergistic effect on the antioxidant property, and the antioxidant property was significantly enhanced, especially when the ratio of Cur/ATH in the intelligent films was 5:5 [41]. An intelligent film with high antioxidant activity could prevent the oxidative degradation of packaged food, which is conducive to the application of food packaging [42].

### 3.11. pH Sensitivity of Bacterial Cellulose Nanofibers/Gelatin-Based Intelligent Films

#### 3.11.1. Color Response of Bacterial Cellulose Nanofibers/Gelatin-Based Intelligent Films at Different pH Values

In order to verify the pH sensitivity of the bacterial cellulose nanofibers/gelatin-based intelligent films, the color changes of the films at different pH values were studied. Figure 7B shows the color changes of bacterial cellulose nanofibers/gelatin-based intelligent films loaded with different Cur/ATH ratios of 10:0, 0:10, 2:8, 5:5 and 8:2 at different pH values (pH = 1, 3, 5, 7, 9, 11 and 13). It could be observed that intelligent films were sensitive to different pH values, which was related to the structural changes of curcumin and anthocyanin, in agreement with a result reported in the literature [23,31]. This result showed that the pH sensitivity of the films was endowed by curcumin and anthocyanin. Furthermore, the color change of the films was similar to that of curcumin and anthocyanin solutions at different pH values, as displayed in Figure 2.

#### 3.11.2. Color Response of Intelligent Films to Volatile Ammonia

**Figure 8:**
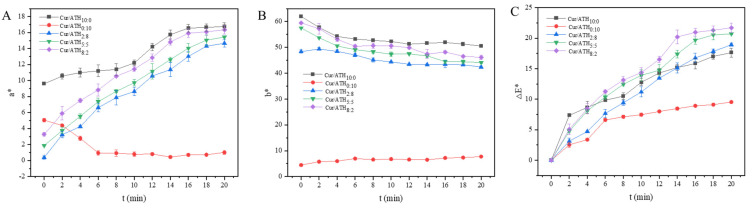
The response of bacterial cellulose nanofibers/gelatin-based intelligent films with different ratios of natural pigments to ammonia vapor at different times: a* value (**A**), b* value (**B**) and ΔE* value (**C**).

In order to simulate the sensitivity of the intelligent films to volatile alkaline compounds generated from meat spoilage, the color response of the intelligent films to volatile ammonia was investigated in the present work. As shown in Figure 8A–C, all of the bacterial cellulose nanofibers/gelatin-based intelligent films were responsive to volatile ammonia. This was mainly due to the attachment of water molecules on the films, which was conducive to the reaction of NH_3_ with H_2_O to form NH_4_^+^ and OH^−^, resulting in the formation of an alkaline environment on the surface of the films, and the resulting OH^−^ could react with the films, thus leading to the color change [43]. With the increase in contact time, the values of ΔE* gradually increased, which was due to the larger concentration of NH_4_^+^ and OH^−^ leading to more obvious color changes. At 20 min, the ΔE^*^ value of the intelligent films loaded with only anthocyanin was the lowest, indicating that anthocyanin was not sensitive to volatile ammonia. As the proportion of curcumin in total pigments increased, the ΔE* value increased from 9.54 (films loaded with a Cur/ATH ratio of 0:10) to 18.93 (films loaded with a Cur/ATH ratio of 2:8), 20.72 (films loaded with a Cur/ATH ratio of 5:5) and 21.72 (films loaded with a Cur/ATH ratio of 8:2) and then decreased to 17.64 (films loaded with a Cur/ATH ratio of 10:0), indicating that the total color difference of the films loaded with curcumin and anthocyanin in response to volatile ammonia was higher than that of the films loaded with only one pigment, especially for the films loaded with a Cur/ATH of 8:2, in agreement with previous reports [44]. This result showed that the mixed pigments were superior for meat freshness detection.

### 3.12. Fresh-Keeping and Freshness Detection Properties of the Bacterial Cellulose Nanofibers/Gelatin-Based Intelligent Films

#### 3.12.1. Storage Quality of the Fresh Pork Packaged with the Bacterial Cellulose Nanofibers/Gelatin-Based Intelligent Films

**Figure 9:**
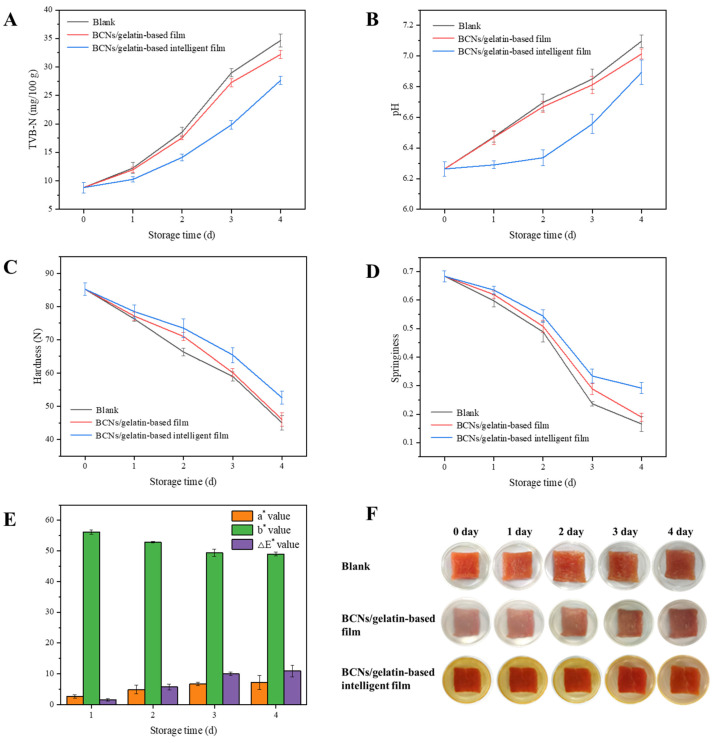
The TVB-N (**A**), pH (**B**), hardness (**C**), springiness (**D**), color (**E**) and appearance (**F**) of pork.

Bacterial cellulose nanofibers/gelatin-based intelligent films with a Cur/ATH ratio of 5:5 were chosen to investigate the fresh-keeping properties of packaging; the quality changes of the fresh pork were evaluated by TVB-N, pH, hardness and springiness. With the decrease in the freshness of pork, microorganisms gradually decompose proteins and produce basic nitrogen-containing toxic substances such as ammonia and amines, which combine with organic acids produced at the same time in the process of meat corruption to form volatile saline base state ammonia existing in meat; as a result, the TVB-N and pH values increase [45,46]. As shown in Figure 9A,B, with the increase in storage time, TVB-N and pH showed an increasing tendency. In the beginning, the TVB-N value was 8.82 mg/100 g, which was lower than the critical value of GB 2707-2016 (fresh meat, 15.00 mg/100 g) [47], indicating that the pork was fresh. On the third day, the TVB-N values of pork without packaging and packaged with bacterial cellulose nanofibers/gelatin films were 28.96 mg/100 g and 27.26 mg/100 g, respectively, exceeding the critical value (spoiled meat, 25.00 mg/100 g) [4], indicating that the pork was completely spoiled. However, the TVB-N value of pork packaged with intelligent films was 19.82 mg/100 g, which was lower than 25.00 mg/100 g, indicating that the pork was relatively fresh and edible. On the third day, the pH values were 6.85 and 6.81 for pork without packaging and packaged with bacterial cellulose nanofibers/gelatin films, respectively, exceeding the critical value (spoiled meat, 6.7), indicating that the pork was spoiled. However, the pH value of pork packaged with intelligent films was 6.56, which was lower than 6.7, indicating that the intelligent films could effectively prolong the shelf life of pork, in agreement with a result in the literature [48].

In addition, the hardness and springiness of pork showed a decreasing tendency during storage (Figure 9C,D) due to the proteins and their hydration layer in meat forming a network structure that has a certain ability to resist external forces. As the storage time increased, the proteins would gradually hydrolyze into some small molecules, the network structure of muscle would be gradually destroyed, and the texture characteristics of meat would decline [18]. On the fourth day, the hardness of pork without packaging and packaged with bacterial cellulose nanofibers/gelatin films was 45.00 N and 46.01 N, respectively, while the hardness of pork packaged with intelligent films was 52.58 N. The springiness of pork without packaging and packaged with bacterial cellulose nanofibers/gelatin films was 0.17 and 0.19, respectively, while the springiness of pork packaged with intelligent films was 0.29. The hardness and springiness of pork with intelligent films were significantly higher than those without packaging and packaged with bacterial cellulose nanofibers/gelatin films, indicating that the intelligent films had the best preservation effect on pork.

#### 3.12.2. Pork Freshness Detection

Nitrogenous compounds produced by the degradation of meat under the action of enzymes and microorganisms increase the pH values of the microenvironment inside a package; thus, intelligent films loaded with natural pigments that display color changes at different pH values could be used for meat freshness detection. In the present work, bacterial cellulose nanofibers/gelatin-based intelligent films loaded with a Cur/ATH ratio of 5:5 were used to detect the freshness of fresh pork. As shown in Figure 9E, the color parameters of the films changed during storage; the a* value increased from 2.61 to 7.26, the b* value decreased from 56.19 to 48.96 and the ΔE* value increased from 1.61 to 10.90. Moreover, the color of the intelligent films changed from yellow to red (Figure 9F), which was due to the accumulation of TVB-N leading to increased pH values, which was confirmed in Figure 9A. The results showed that intelligent films could be used for pork freshness detection [39,49].

## 4. Conclusions

Highly pH-sensitive bacterial cellulose nanofibers/gelatin-based intelligent films loaded with curcumin/anthocyanin were prepared. The results of SEM and AFM showed that the natural pigments significantly affected the morphology and structure of the films. FTIR analysis showed that curcumin and anthocyanin were successfully immobilized in the gelatin matrix. Compared with the intelligent films loaded with only one pigment, the barrier and mechanical properties of the intelligent films loaded with two pigments were significantly improved. The antioxidant and pH sensitivity properties of the bacterial cellulose nanofibers/gelatin-based films loaded with two pigments were better than those of films loaded with only one pigment. In particular, films loaded with a Cur/ATH ratio of 5:5 had the best mechanical and antioxidant properties and a high pH sensitivity property. In addition, bacterial cellulose nanofibers/gelatin-based intelligent films loaded with a Cur/ATH ratio of 5:5 were used for the packaging of fresh pork. The results showed that the quality of pork packaged with the prepared intelligent films was the best, and the color of the intelligent films gradually changed from yellow to red, indicating that the film could be used not only for the preservation of pork, but also for the freshness detection of pork. Therefore, the intelligent film has great potential for the fresh-keeping of meat and meat freshness detection.

## Figures and Tables

**Table 1:** Roughness parameters of bacterial cellulose nanofibers/gelatin-based intelligent films.

Film Type	Rq (nm)	Ra (nm)
Cur/ATH_10:0_	52.75 ± 6.58 ^a^	41.90 ± 7.21 ^a^
Cur/ATH_0:10_	16.70 ± 0.57 ^c^	13.15 ± 0.64 ^c^
Cur/ATH_2:8_	28.85 ± 2.05 ^b^	21.65 ± 0.92 ^bc^
Cur/ATH_5:5_	30.50 ± 2.12 ^b^	21.90 ± 0.57 ^bc^
Cur/ATH_8:2_	34.95 ± 4.17 ^b^	25.55 ± 5.73 ^b^

The data are represented by the mean ± standard deviation. Different letters (a, b and c) differ significantly on the same line.

**Table 2:** Thickness, water content (WC), water solubility (WS), water vapor permeability (WVP) and relative oxygen transmission rate (ROT) of bacterial cellulose nanofibers/gelatin-based intelligent films with different ratios of natural pigments.

Film Type	Thickness (mm)	WC (%)	WS (%)	WVP (10^−10^ g∙m/m^2^∙s∙Pa)	ROT (10^−3^ g/m^2^∙s)
Cur/ATH_10:0_	0.030 ± 0.001 ^a^	8.79 ± 2.66 ^b^	49.12 ± 3.74 ^c^	2.22 ± 0.25 ^bc^	2.96 ± 0.09 ^a^
Cur/ATH_0:10_	0.026 ± 0.002 ^c^	20.82 ± 2.43 ^a^	63.58 ± 2.31 ^a^	2.67 ± 0.19 ^a^	2.81 ± 0.29 ^ab^
Cur/ATH_2:8_	0.027 ± 0.002 ^bc^	17.32 ± 2.65 ^a^	58.00 ± 1.27 ^ab^	2.57 ± 0.22 ^ab^	2.75 ± 0.07 ^ab^
Cur/ATH_5:5_	0.028 ± 0.001 ^ab^	16.88 ± 1.63 ^a^	56.27 ± 0.90 ^b^	2.37 ± 0.04 ^abc^	2.44 ± 0.36 ^b^
Cur/ATH_8:2_	0.028 ± 0.002 ^ab^	14.31 ± 2.74 ^ab^	52.91 ± 1.60 ^bc^	2.09 ± 0.22 ^c^	2.87 ± 0.14 ^ab^

The data are represented by the mean ± standard deviation of three repetitions. Different letters (a, b and c) differ significantly on the same line.

**Table 3:** Tensile strength (TS) and elongation at break (EAB) of bacterial cellulose nanofibers/gelatin-based intelligent films with different ratios of natural pigments.

Film Type	TS (Mpa)	EAB (%)
Cur/ATH_10:0_	45.03 ± 0.36 ^e^	13.25 ± 0.69 ^d^
Cur/ATH_0:10_	52.71 ± 2.64 ^c^	15.30 ± 0.39 ^bc^
Cur/ATH_2:8_	57.01 ± 0.72 ^b^	16.70 ± 0.32 ^ab^
Cur/ATH_5:5_	62.96 ± 1.61 ^a^	17.42 ± 0.73 ^a^
Cur/ATH_8:2_	49.06 ± 1.49 ^d^	14.73 ± 0.75 ^c^

The data are represented by the mean ± standard deviation of three repetitions. Different letters (a, b, c, d and e) differ significantly on the same line.

**Table 4:** Color properties of bacterial cellulose nanofibers/gelatin-based intelligent films with different ratios of natural pigments.

Film Type	L*	a*	b*	ΔE*
Cur/ATH_10:0_	85.30 ± 0.09 ^a^	9.61 ± 0.13 ^a^	61.98 ± 0.44 ^a^	60.92 ± 0.47 ^a^
Cur/ATH_0:10_	85.51 ± 0.76 ^a^	5.05 ± 0.22 ^b^	4.47 ± 0.26 ^e^	11.15 ± 0.80 ^e^
Cur/ATH_2:8_	85.26 ± 0.29 ^a^	0.39 ± 0.20 ^e^	48.37 ± 0.63 ^d^	46.72 ± 0.67 ^d^
Cur/ATH_5:5_	84.79 ± 0.04 ^a^	1.88 ± 0.07 ^d^	57.52 ± 0.26 ^c^	55.83 ± 0.27 ^c^
Cur/ATH_8:2_	85.42 ± 0.33 ^a^	3.28 ± 0.22 ^c^	59.48 ± 0.39 ^b^	57.72 ± 0.39 ^b^

The data are represented by the mean ± standard deviation of three repetitions. Different letters (a, b, c, d and e) differ significantly on the same line.

## Data Availability

The data used to support the findings of this study can be made available by the corresponding author upon request.
